# Removal of chromium from textile wastewater using corncob-derived activated carbon in an industrial case study at MAA garment

**DOI:** 10.1038/s41598-026-47824-9

**Published:** 2026-04-17

**Authors:** Maebele Gereziher Zegeye, Wondalem Misganaw Golie, Asmelash Gebrekidan Mekonen, Desbelom Welegebrial Hagos

**Affiliations:** 1 Department of Chemical Engineering, Ethiopian Institute of Technology-Mekelle, 231 Mekelle, Ethiopia; 2 Department of Chemical Engineering, College of Engineering, Ethiopian Defense University, 1041 Bishoftu, Ethiopia

**Keywords:** Activated carbon, Adsorption, Chromium, Corncob, Wastewater, Sustainability, Chemistry, Engineering, Environmental sciences

## Abstract

Rapid industrialization unleashes toxic hexavalent chromium (Cr(VI)) from textile effluents into waterways, threatening ecosystems and human health in water-stressed regions like Ethiopia. This study harnesses abundant corncob waste to fabricate chemically activated carbon (CCAC) via KOH impregnation and pyrolysis, optimizing Cr(VI) removal from real post-biological wastewater of MAA Garment Factory, Mekelle (initial 1.35 mg/L Cr(VI)). Batch experiments using response surface methodology (central composite design, 29 runs) identified optimal conditions: 0.175 g/L dosage, pH 6.5, 75 min contact time, 200 rpm achieving 99.45% removal (residual < 0.007 mg/L, below WHO/EPA limits) and Langmuir qₘ of 515.5 mg/g (R²=0.999). Pseudo-second-order kinetics and FTIR/SEM confirmed chemisorption via –OH/C = O groups on porous surfaces, outperforming many biomass adsorbents. This low-cost, regenerable solution advances circular economy principles, directly supporting SDGs 6 (clean water), 9 (innovation), and 12 (waste valorization). Pilot scaling and multi-metal tests promise industrial viability for sustainable wastewater polishing.

## Introduction

Rapid industrialization has significantly increased the discharge of hazardous pollutants into aquatic environments, posing serious risks to ecosystems and human health. Among these pollutants, heavy metals are of particular concern due to their toxicity, persistence, and non-biodegradable nature^1,2^. Chromium is extensively used in textile dyeing, electroplating, pigment production, and metal finishing; however, inadequate treatment of chromium containing effluents has resulted in widespread contamination of surface and groundwater resources. Hexavalent chromium (Cr(VI)) is especially problematic because of its high solubility, mobility, and carcinogenicity, and has been linked to severe health disorders and ecological damage^3,4^.

Textile wastewater is generally characterized by a complex chemical composition, including heavy metals, salts, surfactants, and organic compounds like indigo dyes with high COD levels. Biological treatment effectively reduces BOD and COD by degrading these organics, leaving post biological effluents dominated by residual inorganic pollutants such as heavy metals that require polishing treatments to meet discharge standards^5^.

Among the various chromium removal technologies reported, chemical precipitation, coagulation flocculation, membrane filtration, and ion exchange have been widely applied. Nevertheless, these methods often suffer from limitations such as sludge generation, high operational costs, secondary pollution, or reduced efficiency at low contaminant concentrations. In contrast, adsorption has emerged as a robust and versatile treatment technique, particularly suitable for post treatment polishing, due to its simplicity, high efficiency, and adaptability to varying wastewater matrices^6,7^. Recent literature highlights adsorption as a promising approach for removing residual heavy metals from treated industrial effluents.

Biomass derived carbon remains one of the most effective adsorbents for heavy metal removal owing to its high surface area, well developed porosity, and abundance of surface functional groups^8,9^. Among various agricultural residues, corncobs represent an abundant and underutilized biomass resource, their favorable lingo cellulosic composition, low ash content, and high carbon yield make them suitable precursors for activated carbon production^10,11^.

Studies report that chemically activated biomass derived carbons can exhibit exceptionally high adsorption capacities due to enhanced pore development and surface functionality^12,13^. Many adsorption studies still rely on synthetic solutions, which may fail to capture the chemical complexity and regulatory relevance of real industrial effluents. Moreover, limited research has focused on chromium removal from real textile wastewater collected after biological treatment, particularly using locally available agricultural waste materials.

In this context, the present study evaluates the potential of corncob derived activated carbon (CCAC) for the removal of Cr(VI) from real textile wastewater obtained from the final discharge point of the MAA Garment Textile Factory in Mekelle, Ethiopia. The wastewater was comprehensively characterized, with emphasis on heavy metal content, while organic matter was negligible due to prior biological treatment. The study integrates adsorbent synthesis and characterization, batch adsorption experiments, and statistical optimization using response surface methodology (RSM). By addressing residual chromium contamination under realistic post treatment conditions, this work provides an environmentally relevant and sustainable solution aligned with circular economy and water quality protection goals.

Beyond addressing the identified research gaps, this study is aligned with the global agenda for sustainable development and contributes directly to several United Nations Sustainable Development Goals (SDGs). In particular, it supports SDG 6 (Clean Water and Sanitation, Target 6.3) by enabling the removal of a hazardous heavy metal from industrial wastewater, thereby improving effluent quality and reducing risks to aquatic ecosystems and public health. The conversion of agricultural waste (corncobs) into a functional activated carbon further advances SDG 12 (Responsible Consumption and Production, Targets 12.4 and 12.5) through waste valorization, reduced dependence on non-renewable resources, and environmentally sounds management of chemicals. Moreover, the development of a locally sourced and low cost adsorbent contributes to SDG 9 (Industry, Innovation and Infrastructure) by promoting sustainable industrial practices and technological innovation, with indirect benefits to SDG 3 (Good Health and Well-being) and SDG 11 (Sustainable Cities and Communities).

Recent literature increasingly emphasizes the importance of aligning wastewater treatment technologies with the SDG framework, particularly in developing economies where rapid industrialization often places pressure on environmental protection systems^14,15^. Within this context, adsorption-based treatment methods employing waste-derived bio-sorbents have gained significant attention as sustainable alternatives to conventional remediation technologies, offering competitive removal efficiency alongside economic and environmental advantages^16–18^. Comparative studies indicate that such materials can effectively address industrial wastewater challenges while supporting resource recovery and circularity. In parallel, recent research highlights the need to assess sustainability beyond technical performance, incorporating environmental impact, long-term viability, and life-cycle considerations into the evaluation of wastewater treatment solutions^19,20^. The integration of waste-derived adsorbents into circular economy frameworks has been identified as a critical pathway to enhance resource efficiency and minimize secondary pollution in industrial water management systems^21–24^. In this regard, the present study contributes to the growing body of SDG oriented research by proposing a sustainable, locally adaptable approach for chromium removal from real post-biological textile wastewater.

## Materials and methods

### Study area description

**Fig. 1:**
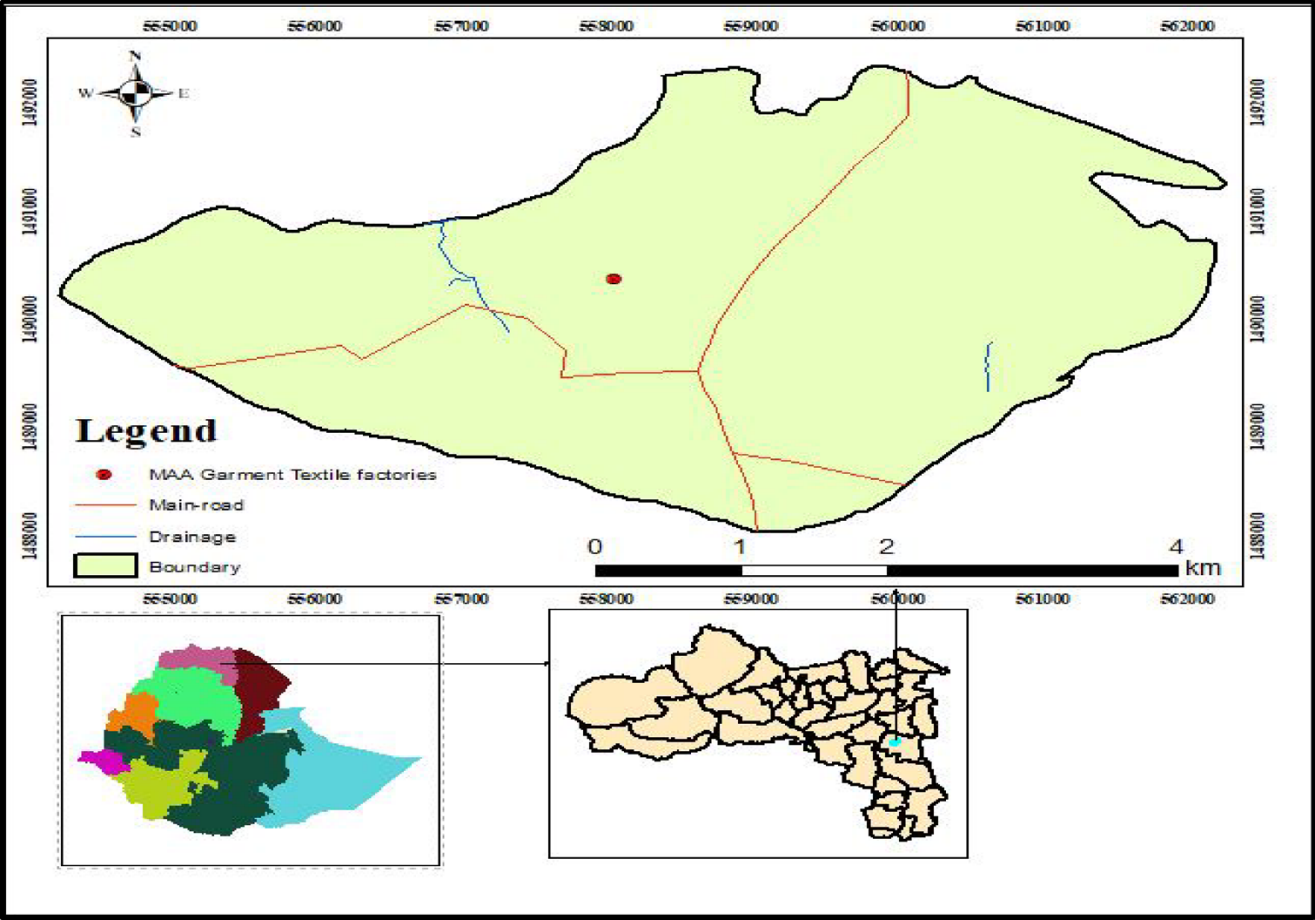
Location of study area. The map was produced using ArcGIS version 10.5 (Esri Inc., Redlands, CA, USA), available at https://www.esri.com/en-us/arcgis/about-arcgis/overview.

The research study was conducted at the MAA Garment and Textile Factory, which is located near Alula Abanega airport, southeast of Mekelle city, Tigray, Ethiopia. The factory’s location is given in Fig. 1. It uses about 24,000 m^3^/day of fresh water and discharges 13,050 m^3^/day of effluents, which accounts for 54.4% of the total effluents.

### Raw material preparation

**Fig. 2:**
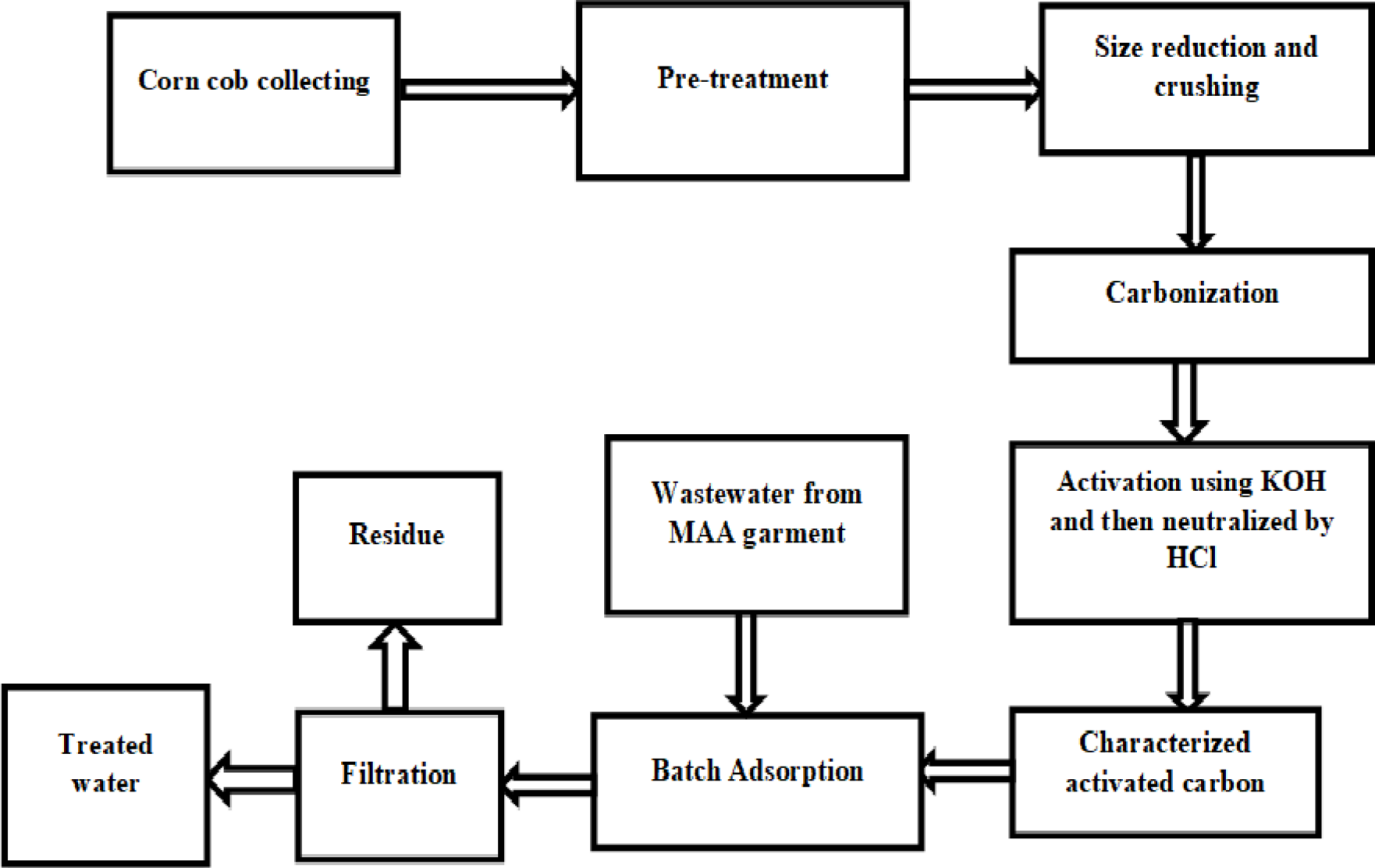
Overall production process of chromium removal from MAA garment wastewater.

Samples were collected from nearby agricultural fields in Quiha, Mekelle. The gathered corn cobs were initially washed with distilled water and then cut into 5 mm pieces. After reducing their size, the resulting pieces were crushed using an electric grinder to achieve a particle size of less than 100 meshes at the Mekelle University Chemical Engineering Laboratory, forming self-adhesive grains. The complete flow diagram for the activation of corncob and adsorption of chromium is given in Fig. 2.

### Carbonization and activation

The grain particles, coated with a self-adhesive substance, were dried before being placed in a furnace heated to 500 °C at a ranging rate of 10 °C/min at the Mekelle University Food Science Department. This heating occurred in a flow of 99.995% pure nitrogen gas at a volume of 150 m^3^/min, and the process lasted for 2 h. After cooling to room temperature, the carbon grains were chemically activated using potassium hydroxide (KOH) at a concentration of approximately 5% of the mass of the self-adhesive carbon grains. For this activation, the self-adhesive carbon grains were soaked in 150 ml of distilled water containing KOH for 25 h at room temperature. Following the soaking period, the mixture was dried under sunlight for 24 h. All these procedures were conducted according to previous studies^25,26^. The activated carbon (AC) was subsequently washed with a 0.1 M hydrochloric acid (HCl) solution, followed by rinsing with hot distilled water. This washing process was repeated multiple times until the pH of the filtrate reached between 6 and 7. Finally, the product was dried in an oven at 105 °C for 24 h and stored in a sealed container to prevent air exposure.

### Raw material characterization

#### Proximate analysis

Proximate analysis involves determining the moisture content, volatile matter, ash content, and fixed carbon content of the biomass.

##### Moisture content (MC)

The moisture content on a dry basis was calculated based on a study in an oven dryer at 105 °C^27^

1 $$MC\left( \% \right) = \frac{{W_{1} - W_{2} }}{{W_{1} }}*100$$

where W_1_ is the initial weight of the sample and W_2_ is the final weight of the corn cob sample after drying.

##### Volatile matter (VM)

The corn cob sample was measured based on the ASTM of E872-82 (2013) procedure.1 g of ground biomass up to < 300 µm was kept inside the furnace at 600 °C and raised 950 °C for 6 min in a closed crucible to obtain volatile matter (VM). The volatile matter (%) was calculated according to Eq. (2)^28^.

2 $${\mathrm{V}}_{{\mathrm{M}}} \left( {{\% }} \right) = \frac{{{\mathrm{W}}_{{\mathrm{i}}} - {\mathrm{W}}_{{\mathrm{f}}} }}{{{\mathrm{W}}_{{\mathrm{i}}} }}{*}100$$

where W_i_ is the initial weight of the sample (g) and W_f_ is the final weight of the sample (g).

##### Ash content

The ash content was assessed through the gravimetric technique following ASTM Standards. Corn cob samples weighing 5 g were placed in crucibles and subjected to a 600 °C furnace for 4 h. Subsequently, the crucibles with the sample were taken out and allowed to cool in desiccators. The weight of the crucible and the sample was then recorded and the percentage of ash was determined using Eq. (3) as stated^29^.

3 $$\% \;{\mathrm{ash}}\;{\mathrm{content}} = \frac{{W_{3} - W_{1} }}{{W_{2} - W_{1} }}*100$$

where W_1_ is the weight of the crucible, W_2_ is the combined weight of the dry crucible and the dry sample of biomass and W_3_ is the weight of the dry crucible along with the ash.

##### Fixed carbon (%FC)

The %FC was calculated using the volatile matter, moisture content, and ash amount by difference according^30^.

4 $$\% FC = 100 - \left( {\% VM + \% Ash + \% MC} \right)$$

where %VM is volatile matter, %Ash is ash content and %MC moisture is content.

#### Ultimate analysis

The ultimate analysis of corn cob biomass waste provides information on the elemental composition of the material. The major elements found in corn cobs are carbon (C), hydrogen (H), oxygen (O), nitrogen (N), and sulfur. The elemental makeup of the sample (including carbon, nitrogen, hydrogen, and sulfur) was analyzed utilizing an EA 1112 Flash CHNS/O-analyzer at the Department of Chemistry, Addis Ababa University. The analysis was conducted with a carrier gas flow rate of 120 ml/min, a reference flow rate of 100 ml/min, an oxygen flow rate of 250 ml/min, a furnace temperature set at 900 °C, and an oven temperature maintained at 75 °C.

### Characterization of corn cob activated carbon

The surface functional groups of CCAC were analyzed using Fourier transform infrared (FT-IR) spectroscopy, which was performed with a Perkin Elmer Model 2000 FTIR spectrometer (USA) at the Addis Ababa University chemistry department. Samples of CCAC were combined with potassium bromide (KBr) and pressed into pellets to record the spectra in the range of 4000 to 400 1/cm. The surface morphology and porosity of CCAC were examined using a scanning electron microscope (SEM), JEOL JSM6460LV (Japan). The point of zero charge (PZC) of the CCAC surface was determined by a solid addition method, with some modifications. The experiment was carried out in a series of stoppered Erlenmeyer flasks, each containing 40 mL of 0.001 M potassium nitrate (KNO_3_) solution and 0.2 g of CCAC. The pH of the solution was adjusted between 2 and 12 by adding 0.1 M hydrochloric acid (HCl) or 0.1 M sodium hydroxide (NaOH). The suspension was shaken for 12 h at 120 rpm in a water bath after which the final pH of the liquid phase was measured. The final pH was plotted against the initial pH, and the PZC was taken as the final pH value in the range where a plateau occurred in the curve. The procedure was repeated using 0.01 and 0.1 M KNO_3_ solutions, and the PZC used was the average of the three values.

### Characterization of MAA garment textile factory wastewater

Wastewater samples (500 mL) were collected from MAA Garment final discharge in plastic bottles and transported to the laboratory at 4 °C. Post-adsorption samples were acidified to pH < 2 with HNO₃ for preservation prior to Atomic Absorption Spectrometry (AAS) analysis at the Geology Department Laboratory, Mekelle University. Cr(VI) concentrations were determined at 357.9 nm wavelength using air-acetylene flame, with calibration standards of 0.5–5.0 mg/L (R^2^ > 0.999) and detection limit of 0.02 mg/L. The general equation for adsorption efficiency is stated in Eq. (5).

5 $$\% Removal = \frac{C0 - Ce}{{C0}}*100$$

where C0 initial concentration, and Ce equilibrium concentration.

### Adsorption process

The experimental setup involved executing the adsorption process in batches. The experiment was conducted in a 50-ml beaker equipped with a magnetic stirrer capable of speeds up to 500 rpm with a minimum increment of 5 rpm. The chromium concentration in the beaker was maintained at a constant level of 1.35 mg per liter for varying doses of the adsorbent, ranging from 0.05 to 0.3 g. The experiments were designed to optimize the adsorbent dose, agitation speed, contact time, and pH of the wastewater. The heavy metal adsorption was studied at a constant chromium VI concentration and a specific dose of corn cob-AC adsorbent, with a fixed pH of the solution. Separate experiments were conducted at constant initial chromium VI concentrations of 1.35 mg per liter for each adsorbent dose of 0.05, 0.175, and 0.3 g. Additionally, the pH of the solution was optimized at a specific contact time and dose by performing experiments with different pH levels (2, 6.5, and 11) under fixed stirring conditions at room temperature for a specific agitation speed (100–300) rpm. Samples were withdrawn at different time intervals and analyzed for chromium VI concentration using atomic absorption spectroscopy. The data obtained were applied to kinetics analysis and equilibrium tests were performed.

### Determination of point of zero charge (PZC)

For understanding the adsorption behavior of ions and molecules onto the surface of an adsorbent, such as corn cob-AC PZC parameter was determined. In this research, the experiment for determining the PZC was conducted using the salt addition method^31^, which is a common method to determine the PZC. First, a series of solutions were prepared with different initial pH values from pH 2 to 11 using an inert electrolyte, NaNO_3_. Then, 0.175 g of corn cob-AC was added to each solution, and the suspensions were allowed to equilibrate for 24 h. Next, the final pH value of each suspension was measured and plotted as a graph of change in pH versus initial pH to determine the optimum pH.

### Adsorption isotherm

The adsorption isotherm describes the relationship between the amount of adsorbate (Cr(VI)) adsorbed per unit mass of adsorbent (corn cob AC) and the equilibrium concentration of adsorbate in the solution at a constant temperature. The most commonly used isotherm models are:

#### Langmuir isotherm

The Langmuir adsorption isotherm model is founded on four fundamental assumptions. Firstly, it assumes that adsorption occurs in a single molecular layer, with no additional molecular coverage. Secondly, it posits that the probability of using each adsorption site is identical. Thirdly, it assumes that the surface of the adsorbent is uniform and consistent. Lastly, it stipulates that the likelihood of a molecule binding to a specific location is independent of whether adjacent spaces have already been occupied by other molecules. These assumptions form the basis for the mathematical derivation and validation of the Langmuir isotherm model, which describes the relationship between the amount of adsorbate adsorbed and the equilibrium concentration of the adsorbate in the solution. Its model is represented in Eq. (5) according to^32^.

6 $$\frac{{c_{e} }}{{q_{e} }} = \frac{1}{{q_{m} k_{l} }} + \frac{{c_{e} }}{{q_{m} }}$$

where C_e_ is the concentration of chromium VI (mg/L) at equilibrium, q_e_ is the amount of chromium VI adsorbed by corn cob AC (mg/g) at equilibrium, q_m_ is the maximum adsorption capacity (mg/g), K_l_ is the Langmuir isotherm constant (L/mg).

#### Freundlich isotherm

This model is more suited to describe physical adsorption events. Because it takes into consideration the unequal distribution of adsorption sites and the ensuing multi-layer adsorption behavior, this empirical equation offers a more accurate depiction of the adsorption process. Its model is as below as stated^33^.

7 $$\ln q_{e} = \ln k_{f} + \frac{1}{{n_{f} }}\ln c_{e}$$

where K_f_ is the Freundlich constant or capacity factor, while 1/n_f_ is the Freundlich exponent.

#### Temkin isotherm

Considers the effects of indirect adsorbate/adsorbent interactions on the adsorption process. Its expression of model is as Eq. (7)^32^

8 $$q_{e} = \frac{RT}{{b_{T} }}\ln k_{T} + \frac{RT}{{b_{T} }}\ln c_{e}$$

where R is the universal gas constant, T is the temperature in kelvin, b_T_ is the Temkin constant related to the heat of adsorption and k_T_ is the equilibrium binding constant.

#### Determination of adsorption isotherm

First, the preparation of Cr(VI) solutions at different initial concentrations of 0.5–1.35 mg/L was employed. Then, 0.175 g of corn cob AC was added to each solution and the mixtures were agitated at 200 rpm and a constant temperature of 25 °C until equilibrium was reached. Following this, the final equilibrium concentration of Cr(VI) in the solution was measured. Finally, the amount of Cr(VI) adsorbed per mass of adsorbent at equilibrium was calculated, and the experimental data was fitted to the Langmuir, Freundlich, and Temkin isotherm models.

#### Adsorption kinetics models analysis

Adsorption is mainly influenced by the adsorbent’s physicochemical properties and the conditions of the system. The pseudo-1st-order model is a kinetic model used to describe the adsorption process. It is expressed mathematically in Eq. (8)^34^.

9 $$\log \left( {q_{e} - q_{t} } \right) = \log q_{e} + \frac{{K_{1} }}{2.303}t$$

where q_t_ represents the amount adsorbed per unit mass of adsorbent at time t (mg/g), q_e_ denotes the amount adsorbed per unit mass of adsorbent at equilibrium (mg/g), k_1_ is the pseudo-1st-order rate constant (min^−1^) and t is the contact time (min). The pseudo-2nd-order rate expression is presented below in linear form as stated in^35^.

10 $$\frac{t}{{q_{t} }} = \frac{1}{{q_{e}^{2} K_{2} }} + \frac{t}{{q_{e} }}$$

where K_2_ is the pseudo-2nd-order rate constant (min^−2^), and the other variables are the same as the pseudo-1st-order. The intra-particle diffusion kinetic, using Eq. (10)^36^.

11 $$q_{t} = t^{0.5} k_{id} + c$$

where q_t_ is the amount adsorbed at time t (mg/g), the k_id_ is the intra-particle diffusion rate constant (mg/g.min^0.5^), and C is the thickness of the boundary layer (mg/g).

#### Procedures to determine adsorption kinetics

First, a Cr(VI) solution of 1.35 mg/L was prepared, and 0.175 g of corn cob-AC was added. Then, the mixture was agitated at 200 rpm and a constant temperature of 25 °C, and samples were collected at different time intervals (15, 30, 45, 60, 75, 90, 105, 120 min). Next, the Cr(VI) concentration in the samples was measured, and the amount of Cr(VI) adsorbed per unit mass of adsorbent at each time point was calculated. Finally, the experimental kinetic data was fitted to the pseudo-first-order, pseudo-second-order, and intraparticle diffusion models.

### Experimental design

The use of biomass waste as an adsorbent for removing chromium from wastewater is a novel approach. This technology employs readily available waste materials to treat chromium-contaminated wastewater. Experiments have been conducted using different concentration levels of biomass waste adsorbents. Corn cob-AC has been utilized as the adsorbent, and the experiments have been designed using Design-Expert 13 software. Process parameters such as pH, adsorbent dose, agitation speed, and contact time have been optimized based on the percentage of chromium removal. Equilibrium studies have been performed using Design-Expert 13 software as well. For the response surface method, the central composite design (CCD) has been chosen in this study. A three-level and four-factor design was applied, and the range and levels are presented in Table 1**.** The full factorial design shows a total of 29 experiments using each adsorbent. All the results obtained in the form of percentage removal have been fed into the DOE software to develop a second-order polynomial model. A quadratic response model has been developed using all the linear, quadratic, and interaction terms, as described in Eq. (11):

12 $$Y = C_{o} + \sum C_{i} X_{i} + \sum C_{ii} X_{ii}^{2} + \sum C_{ij} X_{i} X_{j}$$

where; Y is the predicted response (% chromium removal), C_o_ is the constant coefficient, C_i_ is the linear coefficient, C_ii_ is the quadratic coefficient, C_ij_ is the interaction coefficient X_i_ and X_j_ is the independent variables.

**Table 1:** The range and parameters of the independent variables studied in the research are.

Variable	Unit	Level		
		Low	Medium	High
Adsorbent dosage	G	0.05	0.175	0.3
Contact time	Min	30	75	120
pH	–	2	6.5	11
Agitation speed	Rpm	100	200	300

## Result and discussion

### Proximate and ultimate analysis of corn cob

As shown in Table 2 the corn cob proximate analysis revealed that it comprises 7.95% moisture content, 75.24% volatile matter, 3.76% ash content, and 13.05% fixed carbon. The ultimate analysis revealed the elemental composition, including 51.21% carbon, 5.49% hydrogen, 0.3% nitrogen, and 43% oxygen. The comparison between the corn cob-AC used in the present study and previously studied biomass-derived AC shows that corn cob-AC has a higher fixed carbon content than the activated carbons from other biomass sources, as presented in Table 2.

**Table 2:** Comparison between corn cob activated carbon and biomass-derived activated carbon.

Properties	Measures	Corn cob	Sugarcane bagasse	Cassia Fistula	Heavy oil ash
References		Present study	^37^	^12^	^38^
Proximate analysis	Moisture content %	7.95	6.95	9.99	33
	Fixed carbon%	13.05	12.5	10.125	11.3
	Ash%	3.76	3.5	3.385	7.7
	Volatile mater%	75.24	77.05	76.5	48
Ultimate analysis	Nitrogen%	0.3	5.5	0.86	4.4
	Carbon%	43	42.5	40	49.5
	Hydrogen%	5.49	18	7.41	1.2
	Sulfur%	ND	6	0.07	4
	Oxygen%	51.21	28	51.66	30

**Fig. 3:**
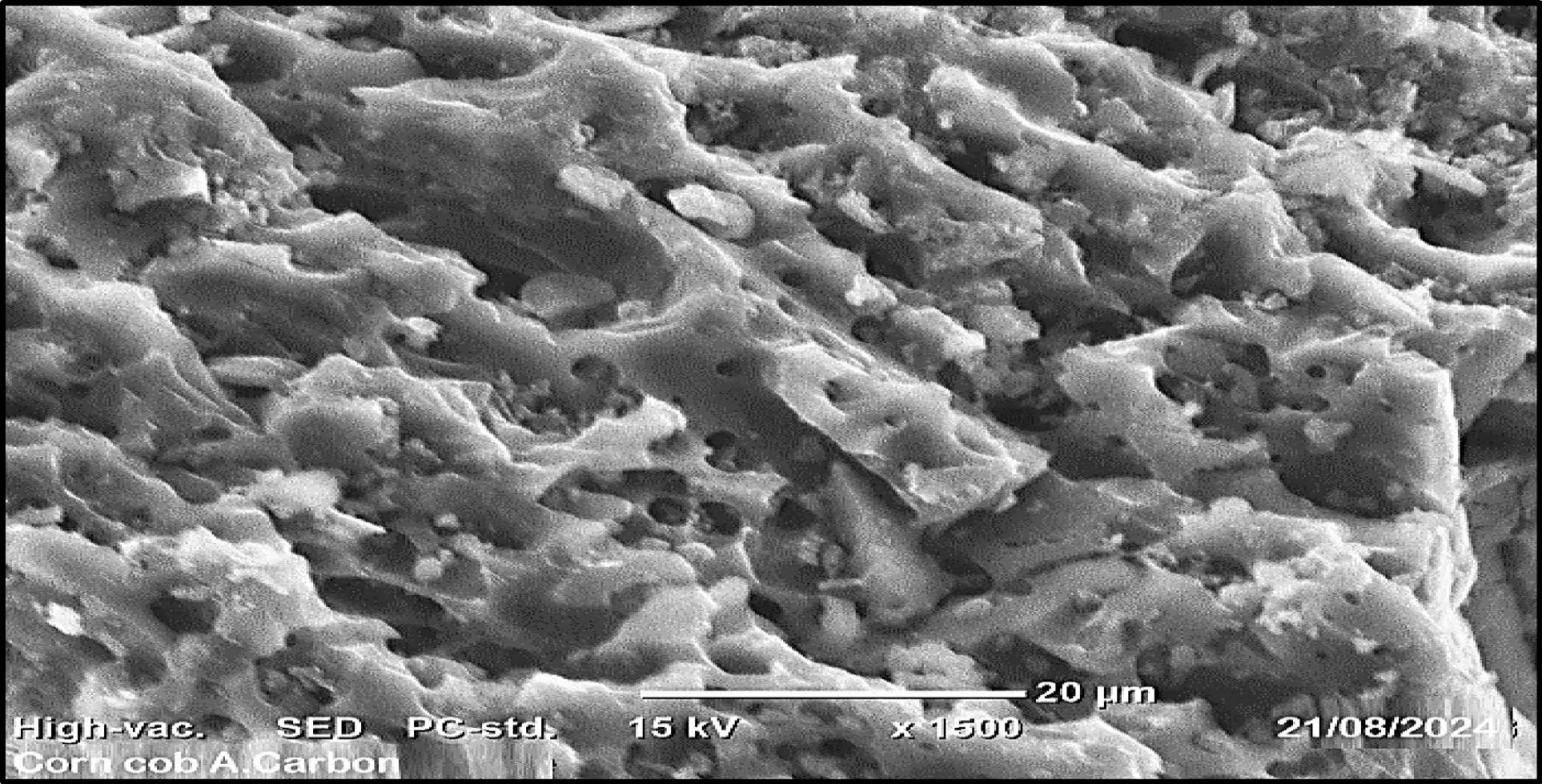
SEM analysis of corncob activated carbon. from the present study.

The scanning electron microscope (SEM) was utilized to analyze the surface structure of porous activated carbon derived from corncobs, as illustrated in Fig. 3. The SEM images reveal a high degree of crystallinity in the corncob-activated carbon (CCAC), which is consistent with previous studies^39^. The primary difference observed is the resolution of the SEM images. CCAC exhibited a distinct two-dimensional layered morphology. Large numbers of macro pores (diameter > 50 nm) with irregular circular shapes were visible in the SEM images of CCAC. Additionally, the rough surface of CCAC suggested the presence of meso pores (2 nm < diameter < 50 nm) and micropores (diameter < 2 nm)^28^. This porous structure allows solutions to penetrate the interior of the AC, providing more adsorption sites for the removal of heavy metals^40^.

### FT-IR analysis

**Fig. 4:**
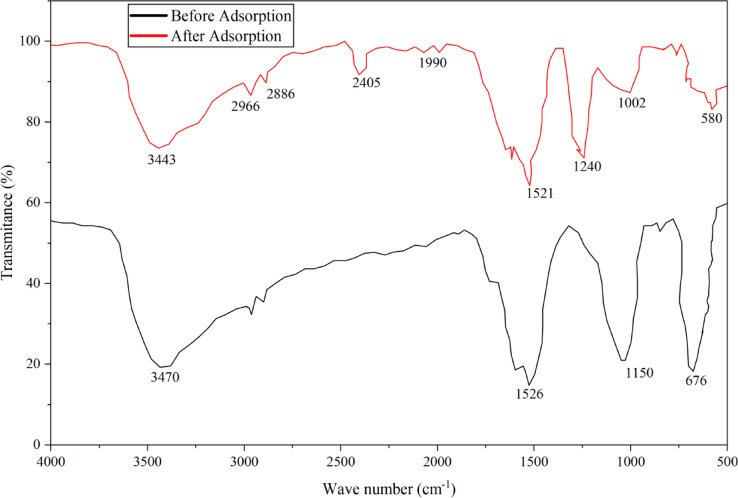
FT-IR spectra of corn cob activated carbon before and after adsorption.

To explore the surface chemistry of the sample, Fourier Transform Infrared Spectroscopy (FTIR) was employed to identify various peaks and functional groups present. The FTIR spectra for the adsorbent before and following the adsorption process are illustrated in Fig. 4. This figure indicates that certain peaks have either shifted or disappeared, while new peaks have emerged, likely as a result of hexavalent chromium adsorption. The FTIR spectra were measured in the wave number regions of 400–4000 cm^−1^. This indicates that various functional groups play a role in the adsorption of chromium. The absorption band observed between 3200 and 3600 cm^−1^ corresponds to the O–H stretching vibrations, suggesting that water molecules are adsorbed onto the bio-sorbent structure^41^. This broadband may also result from the overlapping stretching vibrations of O–H and N–H functional groups. The peak around 3000–2800 cm^−1^ is associated with alkyl groups, specifically the asymmetric C–H stretching^42^. Additionally, the band near 1612 cm^−1^ is linked to the stretching vibrations of C=O carbonyl groups^43^. Another broadband with a peak at approximately 1000–1300 cm^−1^ also pertains to C–O carbonyl groups. The peaks around 2405, 1990, and 1521 cm^−1^ are likely unrelated to corncob-AC and may indicate the presence of minor impurities^42^. Following the adsorption of chromium, a distinct peak observed near 580 cm^−1^ is linked to the presence of chromium on the corncob biosorbent. This enhancement may result from the interaction of metal oxides with potassium hydroxide on the AC derived from corncobs^39^. These observations align closely with^41–43^ findings reported for activated corncob carbon under various activation treatments. Overall, the FT-IR data obtained indicates that AC derived from corncobs is rich in surface functional groups.

**Fig. 5:**
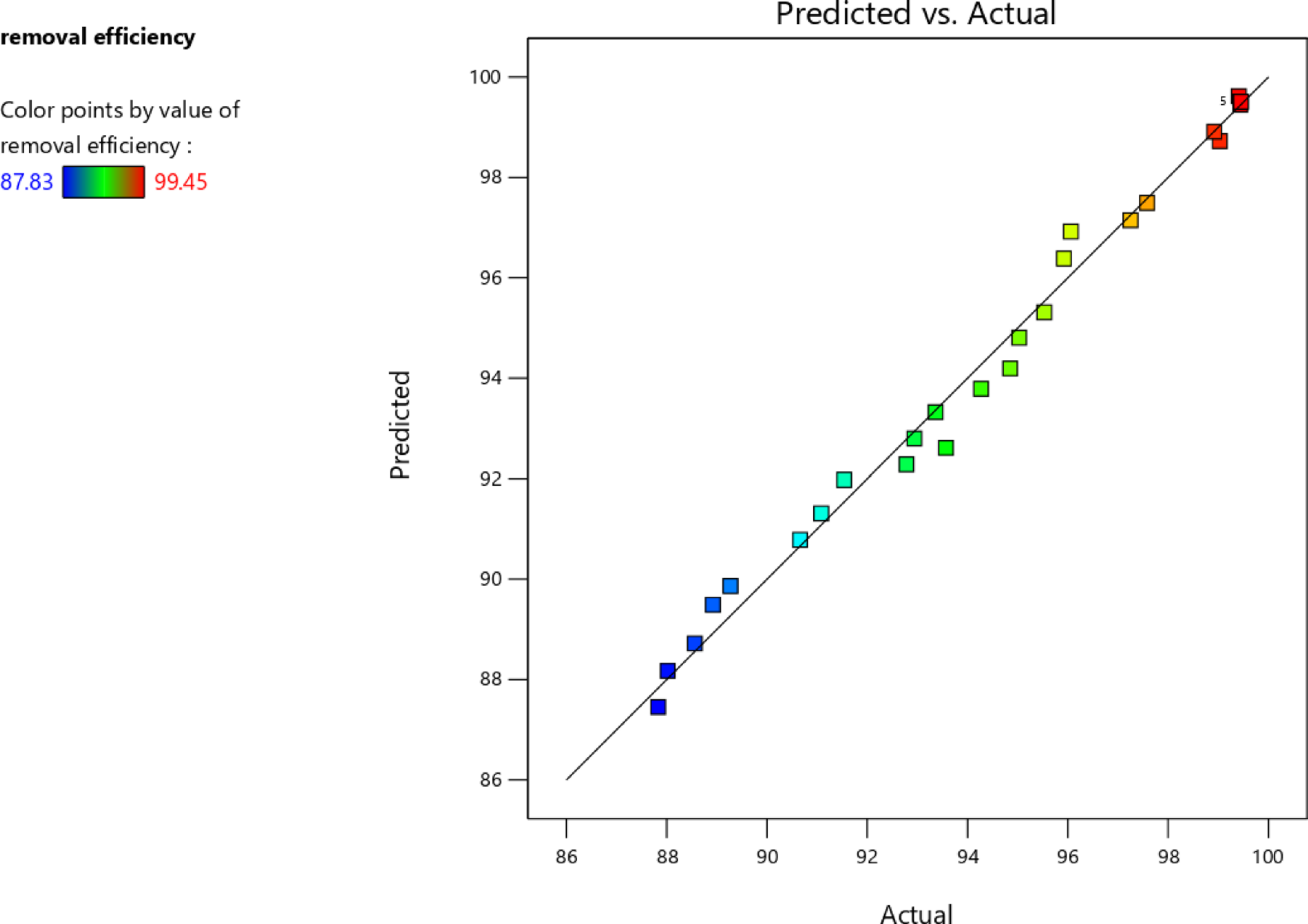
Predicted versus actual values.

### Wastewater characterization

Table 3 presents the results of atomic absorption spectrometry (AAS) conducted on wastewater from the MAA Garment Textile Factory, emphasizing the concentrations of various heavy metals found in the effluent. This data is particularly significant when compared to the maximum allowable limits set by the World Health Organization (WHO) for heavy metals in environmental discharges.

**Table 3:** Heavy metals from MAA garment wastewater with comparison of WHO limit.

Particular	Concentration (mg/l)	WHO maximum limit (mg/l)
Chromium	1.35	0.05
Lead	0.12	0.01
Cd	0.018	0.003
Copper	0.07	0.05
Manganese	0.01	5
pH	8.25	5.5–9.0

The findings reveal critical insights into the levels of contaminants such as lead, cadmium, chromium, Manganese and copper, which are known for their toxic effects on both human health and ecosystems. This comparison underscores the importance of stringent monitoring and regulation of industrial wastewater to mitigate harmful effects on surrounding communities and natural habitats.

### Adsorption results of different treatments

The Central Composite Design for experimental runs using activated corncob carbon to investigate optimum conditions of chromium adsorption, with tested variables like adsorbent dosage, 0.05 to 0.3 gm; contact time, 30 to 120 min; pH, 2 to 11; and agitation speed, 100 to 300 RPM, is presented in the table below. The removal efficiencies are within the range of 87.83% to 99.45%; the highest being attained for only certain combinations of factors. This design identifies key conditions affecting chromium removal and provides a basis for further research and application in wastewater treatment the results are given in Table 4.

**Table 4:** central composite design (CCD) for the optimized chromium adsorption utilizing activated corncob carbon.

Run	Factor 1	Factor 2	Factor 3	Factor 4	Response 1
	A: adsorbent dosage	B: contact time	C: pH	D: agitation speed	Removal efficiency
	Gm	Min		Rpm	%
1	0.175	75	6.5	300	98.92
2	0.175	75	6.5	200	99.45
3	0.05	120	2	100	92.94
4	0.175	30	6.5	200	99.03
5	0.3	120	11	300	92.78
6	0.05	120	11	100	88.02
7	0.3	120	11	100	90.66
8	0.175	75	2	200	96.06
9	0.175	75	6.5	200	99.45
10	0.175	75	11	200	93.57
11	0.05	30	2	300	93.36
12	0.05	120	2	300	94.85
13	0.3	30	2	300	95.53
14	0.05	120	11	300	88.92
15	0.175	75	6.5	200	99.45
16	0.05	30	11	100	87.83
17	0.175	75	6.5	200	99.45
18	0.3	75	6.5	200	99.44
19	0.05	30	2	100	91.54
20	0.3	120	2	100	95.03
21	0.3	30	11	300	91.08
22	0.3	30	2	100	94.27
23	0.3	120	2	300	95.92
24	0.175	120	6.5	200	99.41
25	0.05	30	11	300	88.56
26	0.175	75	6.5	100	97.58
27	0.3	30	11	100	89.27
28	0.05	75	6.5	200	97.25
29	0.175	75	6.5	200	99.45

### Analysis of variance

The ANOVA results in Table 5 indicate that the model is statistically significant (*p* < 0.0001) with a total sum of squares of 450.36. Key factors influencing the model include adsorbent dosage (A) with an F-value of 79.16 (*p* < 0.0001), contact time (B) with an F-value of 11.99 (*p* = 0.0038), pH (C) with an F-value of 278.00 (*p* < 0.0001), and agitation speed (D) with an F-value of 30.14 (p < 0.0001). Interaction effects between factors are not significant, while the quadratic effects for adsorbent dosage (A^2^, *p* = 0.0032) and pH (C^2^, *p* < 0.0001) are significant. The residual sum of squares is 4.21, with no significant lack of fit. Generally, specific factors significantly impact the model’s performance.

**Table 5:** ANOVA for the response removal efficiency.

Source	Sum of squares	df	Mean square	F-value	*p*-value	
Model	450.36	14	32.17	106.87	< 0.0001	Significant
A-adsorbent dosage	23.83	1	23.83	79.16	< 0.0001	
B-contact time	3.61	1	3.61	11.99	0.0038	
C-pH	83.68	1	83.68	278.00	< 0.0001	
D-agitation speed	9.07	1	9.07	30.14	< 0.0001	
AB	0.0400	1	0.0400	0.1329	0.7209	
AC	0.3600	1	0.3600	1.20	0.2926	
AD	0.0324	1	0.0324	0.1076	0.7477	
BC	0.0100	1	0.0100	0.0332	0.8580	
BD	0.0025	1	0.0025	0.0083	0.9287	
CD	0.0064	1	0.0064	0.0213	0.8861	
A^2^	3.79	1	3.79	12.59	0.0032	
B^2^	0.2908	1	0.2908	0.9662	0.3423	
C^2^	58.11	1	58.11	193.04	< 0.0001	
D^2^	4.41	1	4.41	14.64	0.0019	
Residual	4.21	14	0.3010			
Lack of fit	4.21	10	0.4214			Not significant
Pure error	0.0000	4	0.0000			
Cor total	454.58	28				

**Fig. 6:**
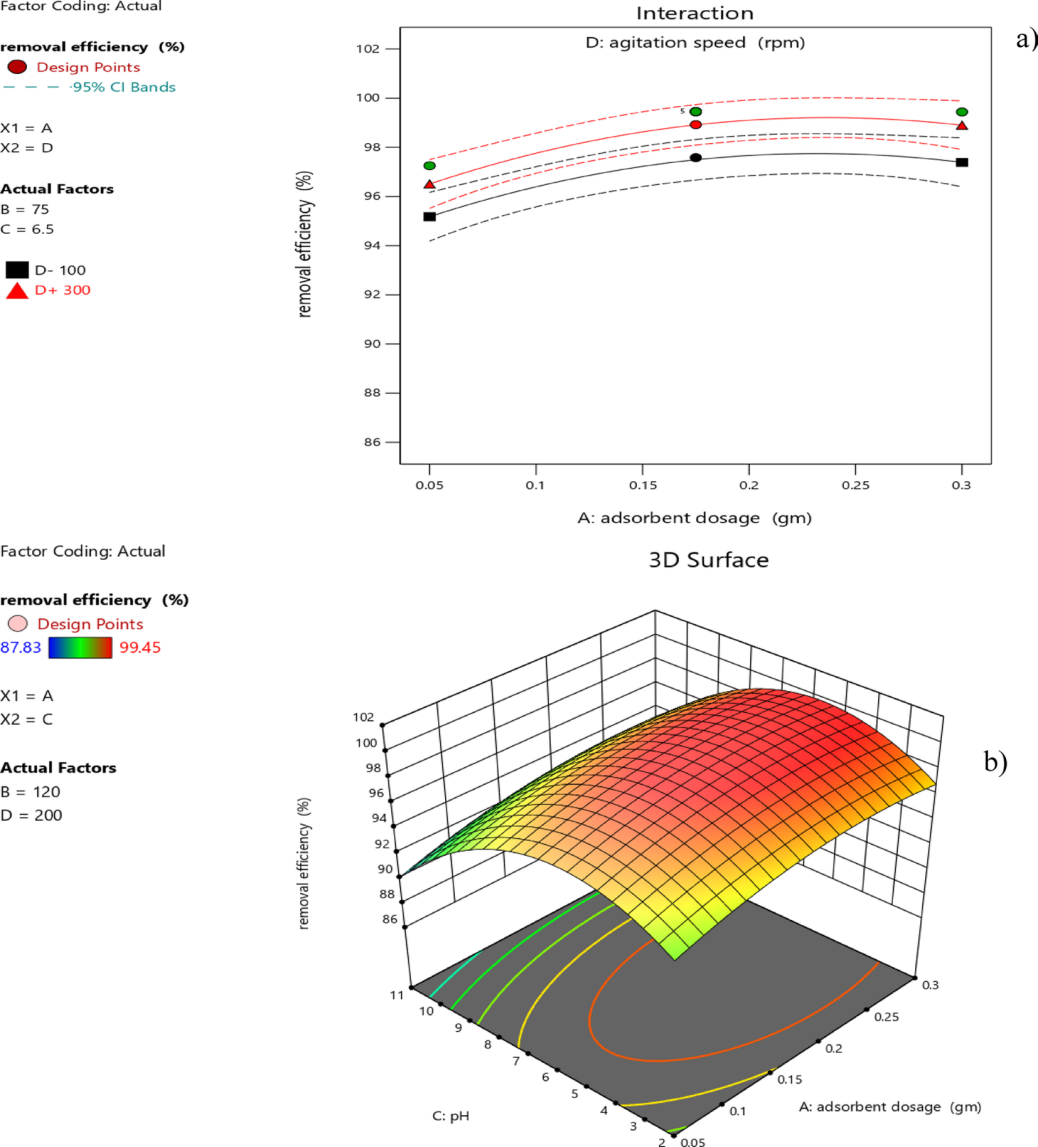
Adsorption effect of chromium (**a**) dosage and agitation speed (**b**) dosage and pH obtained using Design-Expert software (Version 13, Stat-Ease, Inc., Minneapolis, MN, USA), available at https://www.statease.com/software/design-expert/

Figure 6 presents a critical analysis of the correlation between the predicted values generated by the experimental model and the actual observed outcomes in the study of chromium removal from textile wastewater. The x-axis illustrates the predicted values, while the y-axis displays the actual measurements, allowing for a straightforward comparison.

### Effect of adsorbent dosage, contact time, pH and agitation speed

The efficiency of chromium removal using corn cob-AC was evaluated by considering the initial chromium concentration. The results were then analyzed using Design of Experiment (DOE) techniques to establish a statistical model. This model describes the relationship between the dependent variable (chromium removal percentage) and the independent variables (adsorbent dosage, pH, contact time, and agitation speed).

13 $$\begin{aligned} \% removal = & + 80.9 + 33.2A + 0.03B + 2.54C + 0.06D + 0.009AB + 0.27AC \\ & \quad + 0.004AD - 0.0001BC - 77.5A^{2} - 0.0002B^{2} - 0.23C^{2} - 0.0001D^{2} \\ \end{aligned}$$

where A (adsorbent dosage), B (contact time), C (pH), and D (agitation speed).

**Fig. 7:**
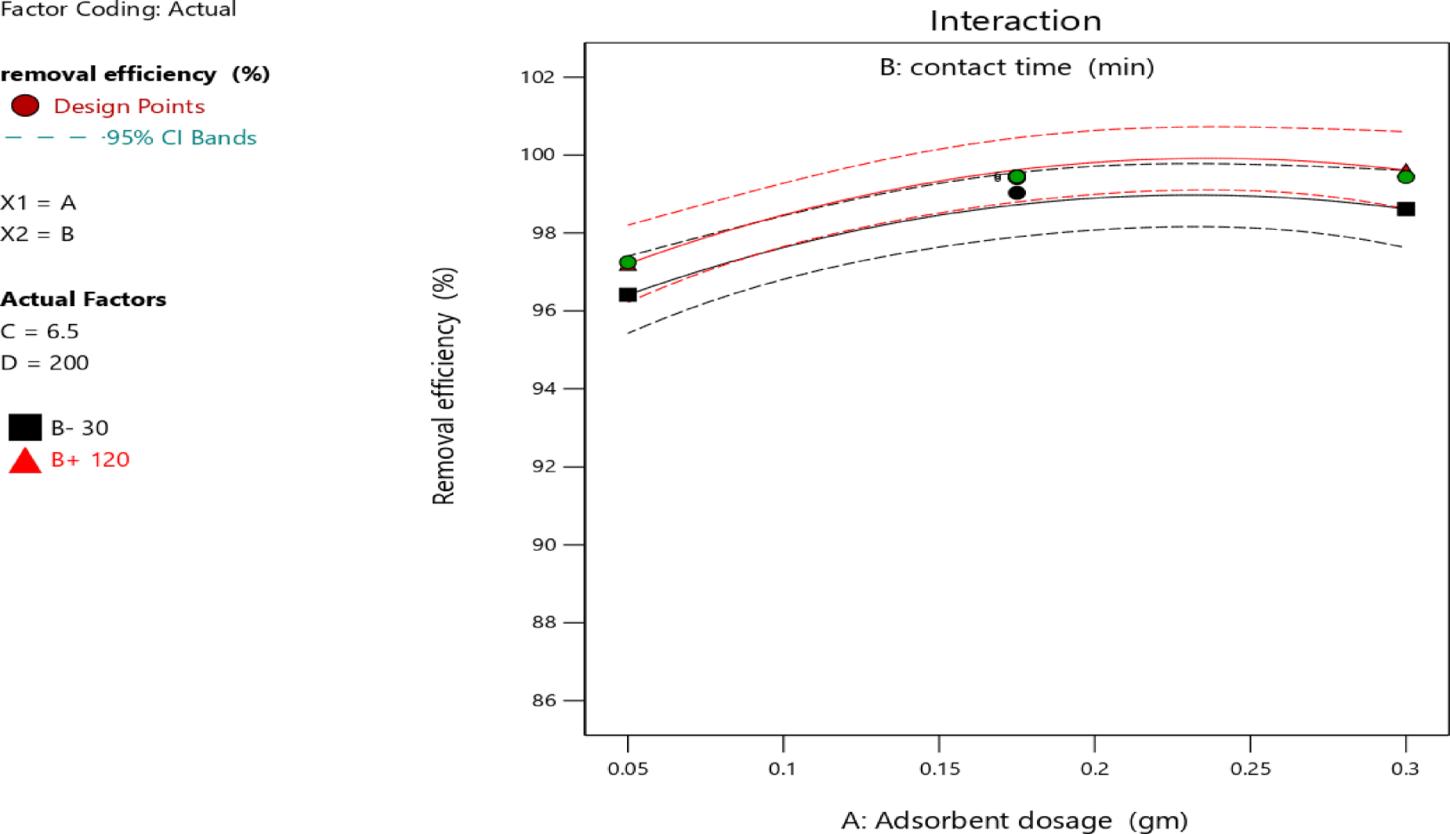
The effect of time and dosage on Adsorption of chromium obtained using Design-Expert software (Version 13, Stat-Ease, Inc., Minneapolis, MN, USA), available at https://www.statease.com/software/design-expert/

**Fig. 8:**
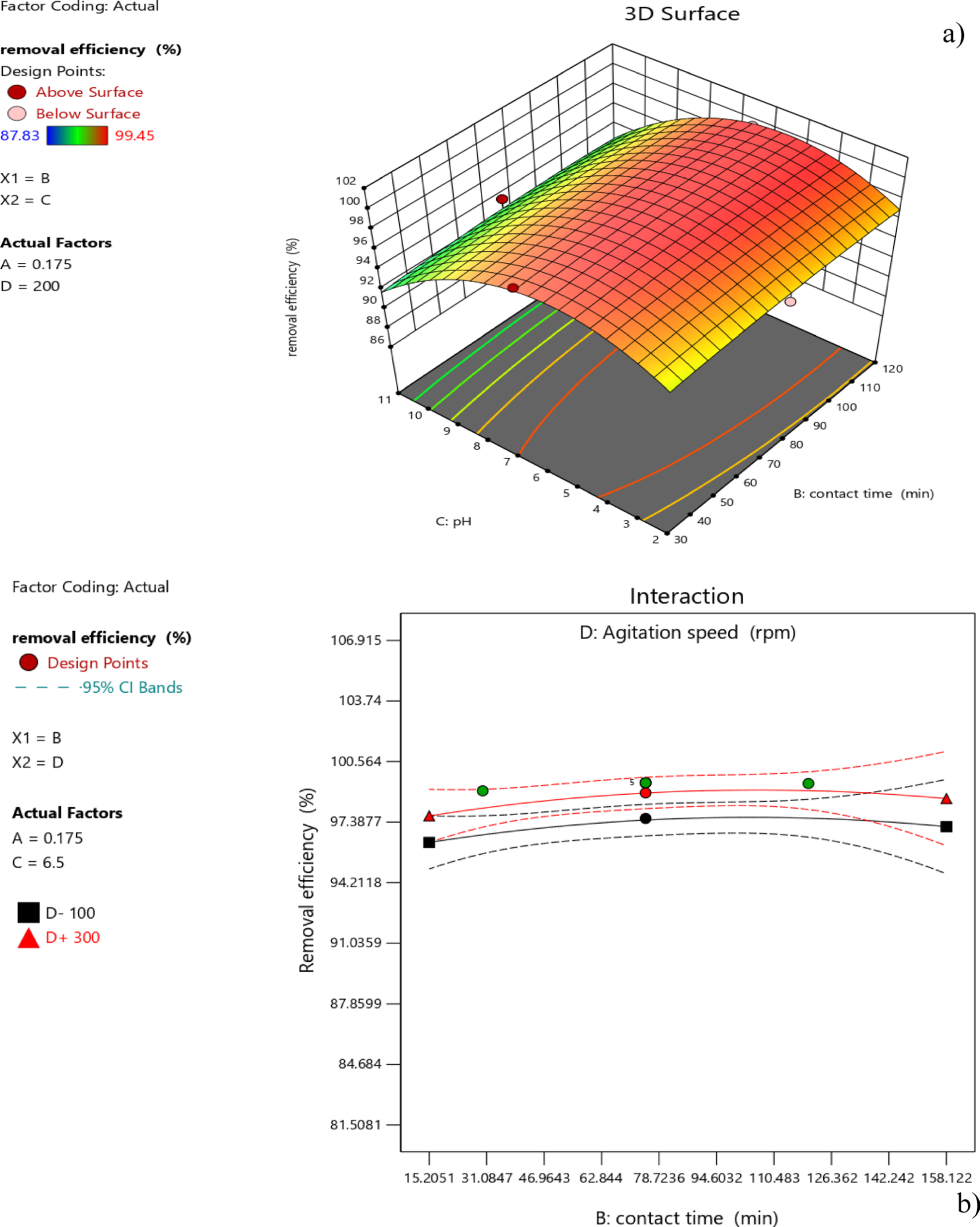
Adsorption effect of chromium (**a**) time and pH (**b**) agitation speed and time obtained using Design-Expert software (Version 13, Stat-Ease, Inc., Minneapolis, MN, USA), available at https://www.statease.com/software/design-expert/

**Fig. 9:**
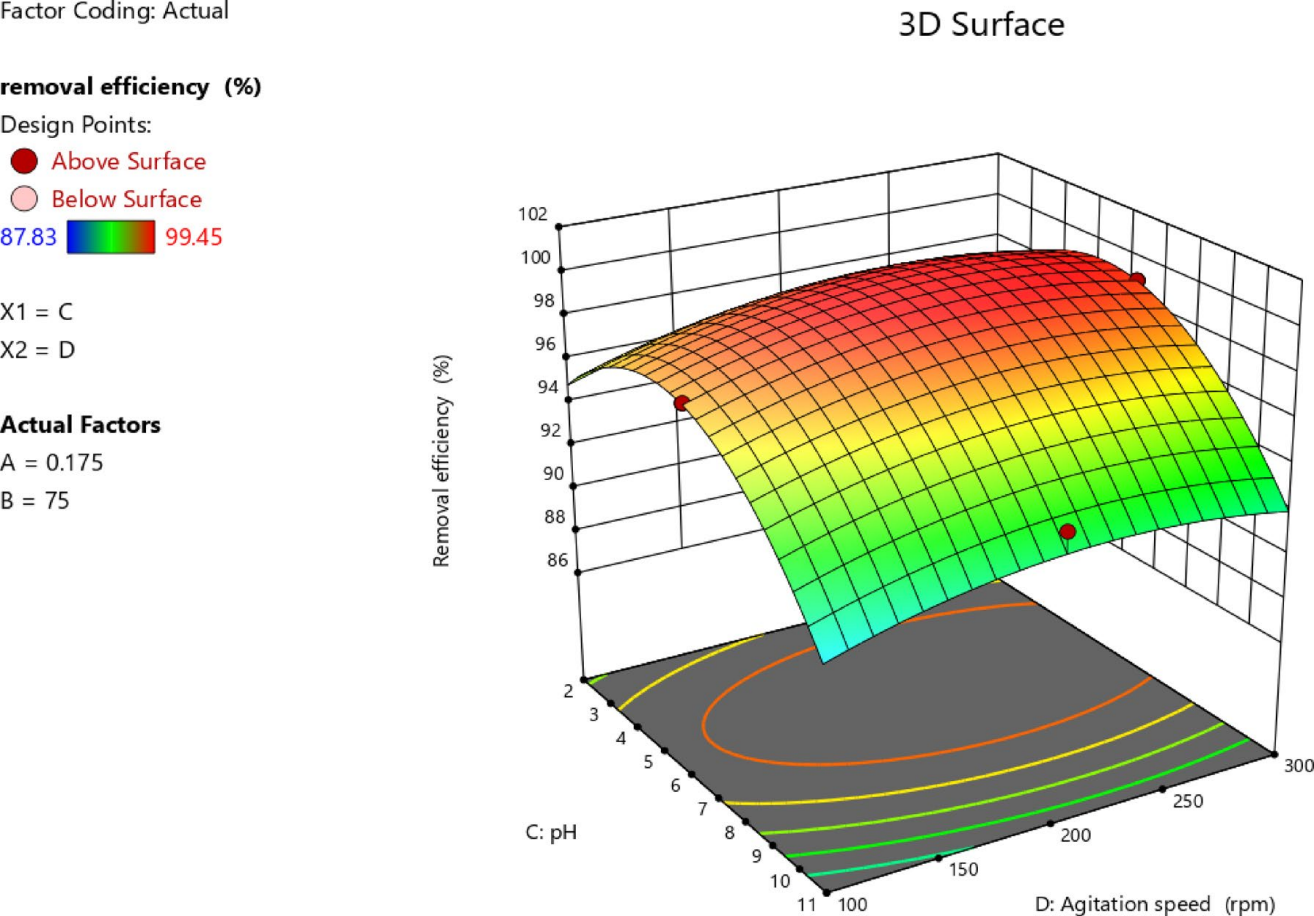
The effect of pH and agitation speed on Adsorption of chromium obtained using Design-Expert software (Version 13, Stat-Ease, Inc., Minneapolis, MN, USA), available at https://www.statease.com/software/design-expert/

As given in Table 5, the ANOVA results show that this model is statistically significant, and it explains a substantial amount of response variable variability. The key factors, all at a very considerable level (*p* < 0.0001), are adsorbent dosage, A; pH, C; and agitation speed, D. Contact time, B, was significant at *p* = 0.0038. Interaction effects among factors were not significant; hence, the factors operate independently. Significant quadratic effects for A and C showed nonlinear relationships. Generally speaking, adsorbent dosage, pH, and agitation speed are the most critical factors while interactions and some quadratic effects are less important. The removal efficiency of chromium (VI) using corncob-derived activated carbon (CCAC) is significantly influenced by key factors, including dosage, contact time, pH, and agitation speed, as well as their interactions. A clear positive correlation exists between CCAC dosage and chromium removal efficiency, as shown in Fig. 7a, B, and C peaking at approximately 0.175 g/L, which enhances adsorption due to increased surface area and availability of adsorption sites, aligning with previous studies highlighting the properties of AC as effective adsorbents^44^. Contact time also plays a critical role, with rapid increases in efficiency observed within the first 30 min, reaching optimal removal at around 75 min as revealed in, Figs. 9a, 7b, and 8. The pH of the solution is another vital factor; maximum removal efficiency occurs at an acidic pH of about 6.5 as represented in Figs. 7a, 9b, and Fig. 8, where chromium species are more readily adsorbed onto the positively charged surface of CCAC. At lower pH levels, chromium(VI) exists mainly as HCrO_4_^−^ and Cr_2_O_7_^2−^, which are more readily adsorbed onto the positively charged surface of the AC. Conversely, at higher pH levels, the surface charge of the AC becomes more negative, reducing the electrostatic attraction for the negatively charged chromium species ^45^.

**Fig. 10:**
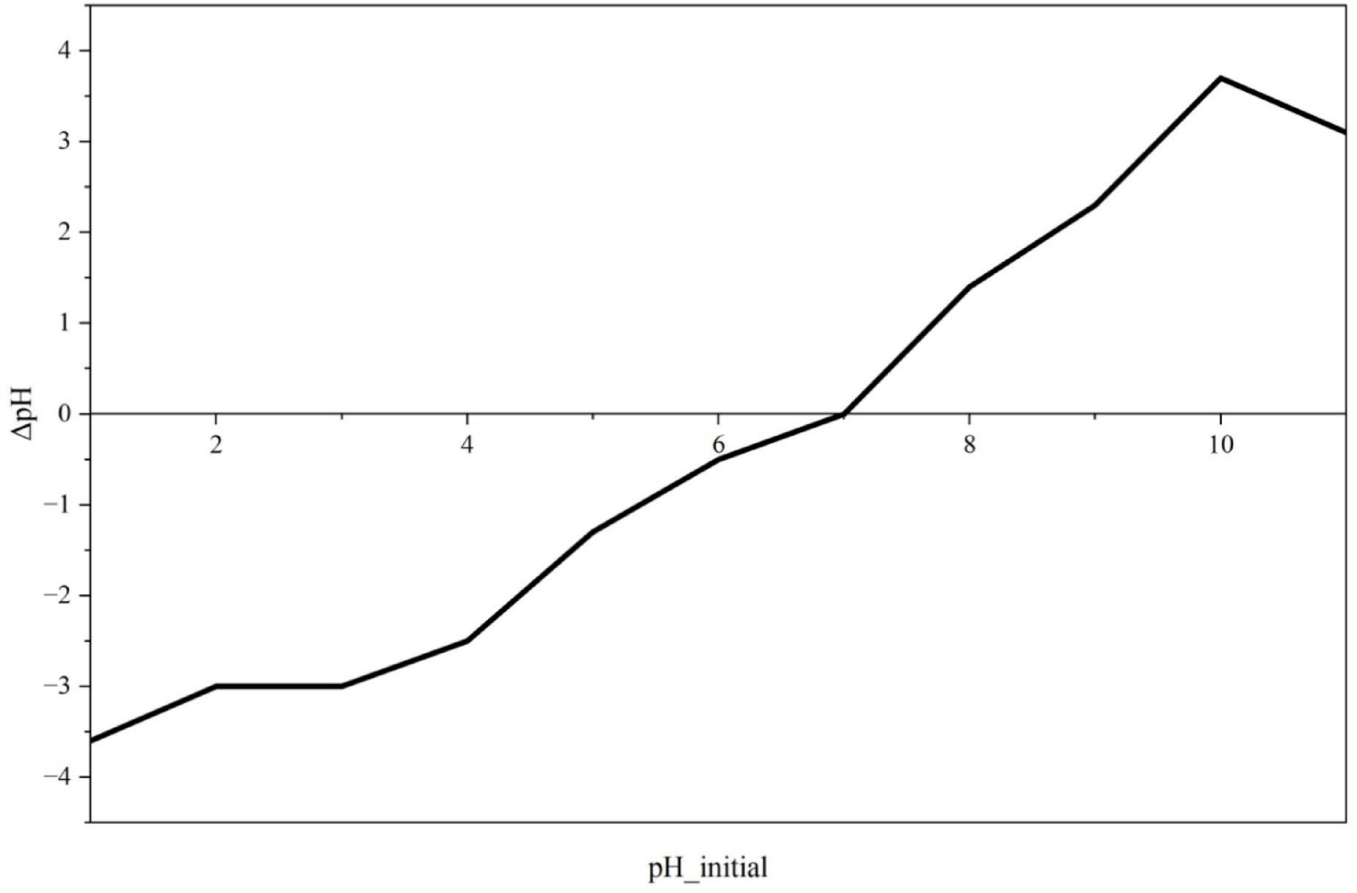
Initial pH versus changes in pH.

Agitation speed further influences efficiency, with optimal mixing achieved at around 200 rpm, beyond which additional agitation yields diminishing returns as shown in Figs. 7a, 8 and 10. The interactions between these factors are complex; for instance, optimal combinations of dosage and agitation speed yield maximum efficiency, while increased contact time enhances removal across all dosages. Additionally, the effectiveness of dosage is most pronounced at lower pH levels, and maintaining an optimal pH is crucial for maximizing the benefits of extended contact times^46^.

### Point of zero charge

**Fig. 11:**
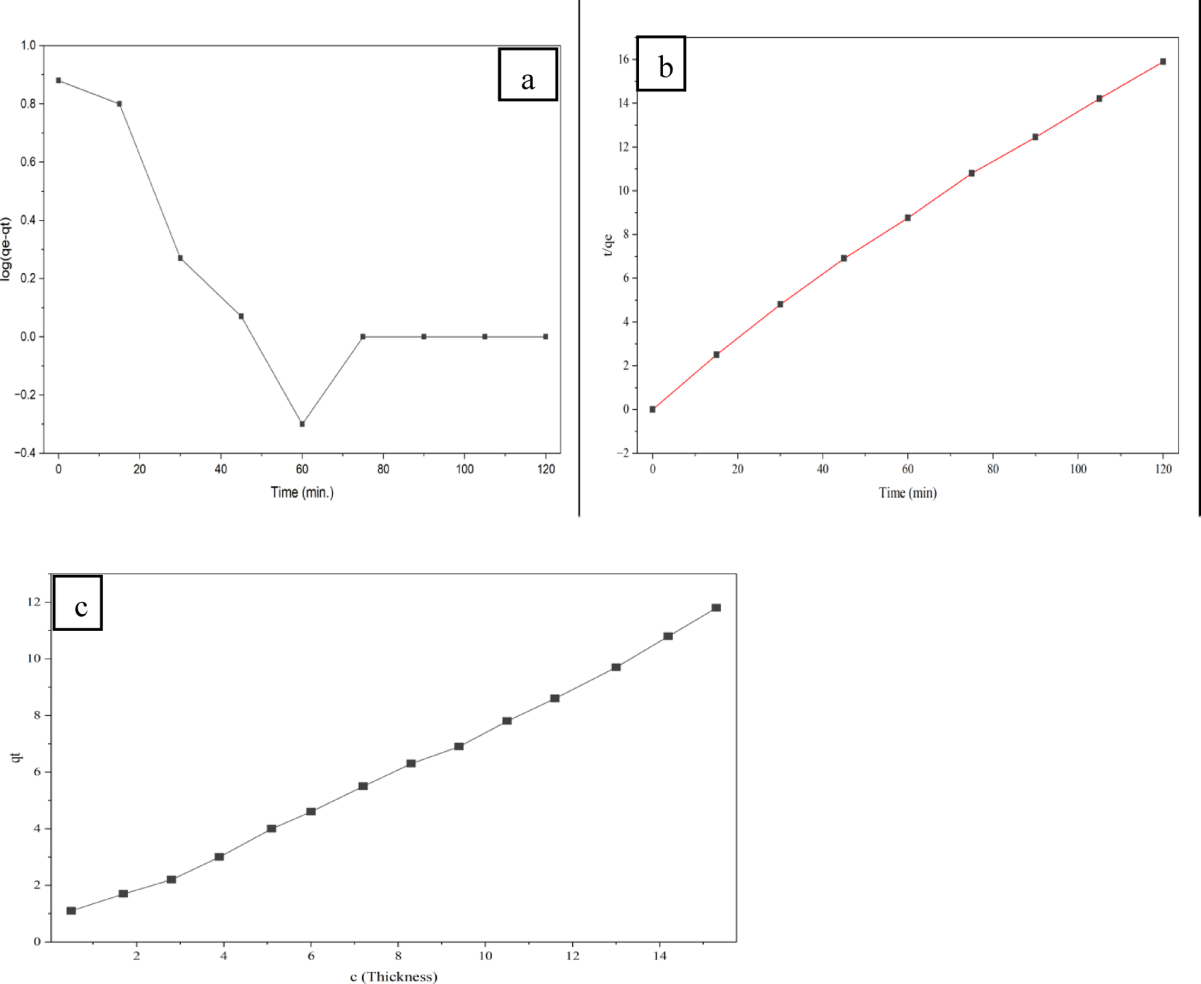
Adsorption kinetics for: Pseudo-first order (**a**), Pseudo-second order (**b**), and Intra particle diffusion (**c**).

At pH values below the PZC, the adsorbent surface is charged positively, favoring the adsorption of negatively charged chromium (VI) species. Conversely, at pH values above the PZC, the adsorbent surface becomes charged negatively^31^, which may lead to electrostatic repulsion between the adsorbent and chromium (VI) ions. Figure 11 shows a graphical representation of the point of zero charge.

### Adsorption kinetics

**Fig. 12:**
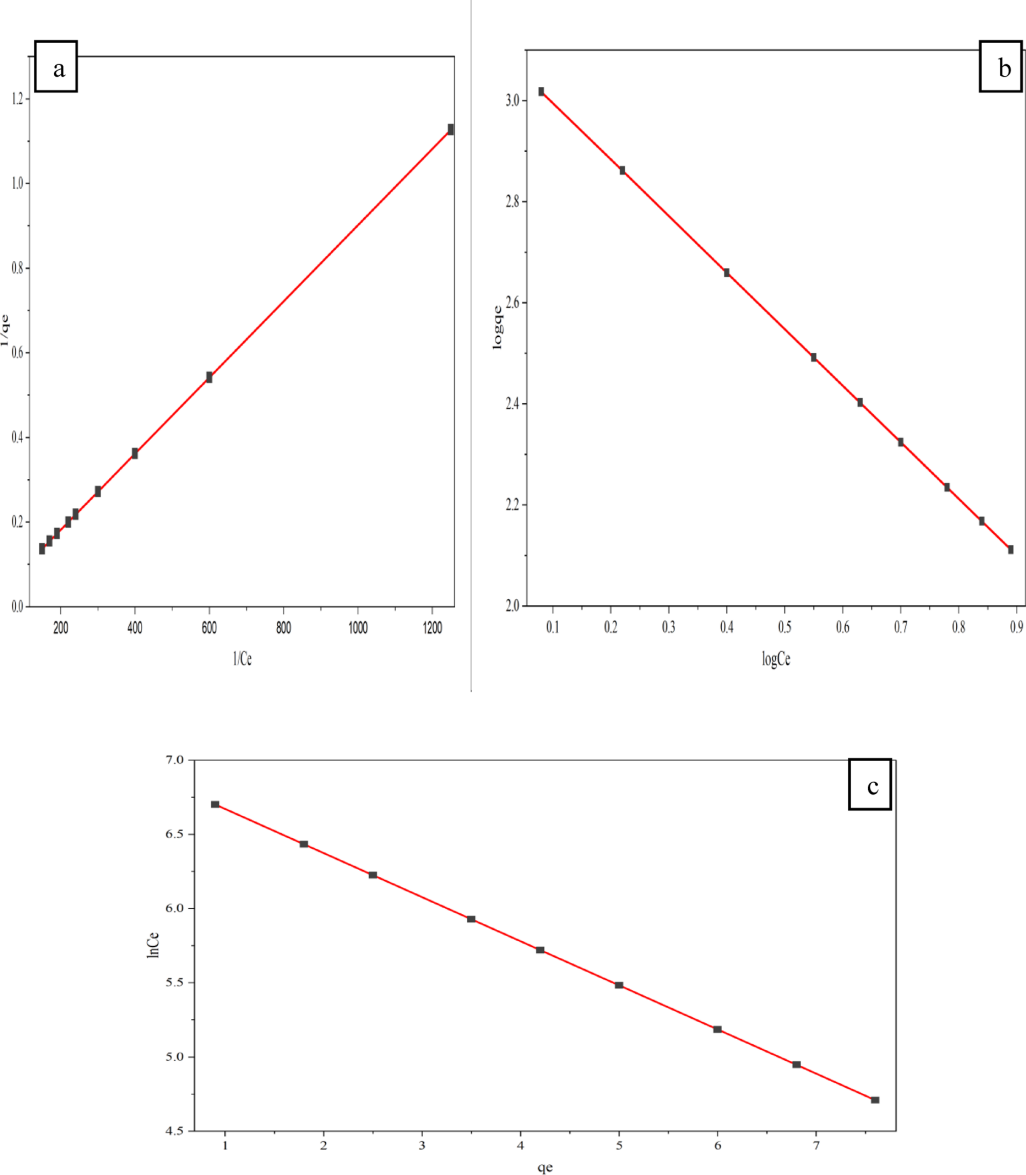
Adsorption isotherm for; Langmuir model (**a**) Freundlich model (**b**) and Temkin model (**c**).

The kinetics of adsorption utilizing AC derived from corn cob biomass waste are illustrated in Fig. 12a–c. The results indicate rapid adsorption of chromium initially, reaching about 95% within the first 60 to 70 min, followed by a gradual increase to a final adsorption rate of 99.45% at a dosage of 175 mg, a pH of 6.5, and an initial concentration of 1.35 mg/L. The corn cob-AC achieved a steady state in 75 min; however, the experiment was extended to 120 min at 25 °C, maintaining a pH of 6.5, to confirm equilibrium adsorption. In this investigation, three distinct kinetic models were employed to analyze the kinetics of chromium (VI) adsorption onto the corn cob-AC. Similar model testing has been previously reported^47^. The models include the pseudo-first order, pseudo-second order, and intra-particle diffusion models, each expressed as linear equations in Eqs. 8–10, respectively.

Table 6 demonstrates that the pseudo-first-order, pseudo-second-order, and intra-particle diffusion models effectively fit the experimental data, exhibiting high correlation coefficients. The corn cob AC (CCAC) adhered to the Langmuir adsorption model, as indicated by a regression coefficient of R = 0.999 for the linear fitted data. The kinetics of chromium (VI) adsorption were best described by the pseudo-second-order model, which had a correlation coefficient of R = 0.987. The maximum adsorption capacity of the CCAC adsorbent was 515.5 mg/g, while the equilibrium adsorption capacity was recorded at 7.67 mg/g. Table 6 compares the kinetic parameters of different models and their regression constants, revealing superior values in this study relative to previous research.

**Table 6:** Comparative studies of the kinetic parameters derived from corn cob.

Kinetic model	Parameters	Corn cob	Mango peel	Groundnut shell	Junk fruit peel
Pseudo-first order	K_1_ (min^−1^)	0.0078	0.095	0.06	0.38
	q_e_ (mg/g)	1.99	5.21	2.76	8.03
	R^2^	0.912	0.97	0.6	0.44
Pseudo-second order	K_2_ (g/mg min)	0.005	0.0054	9.16	0.11
	q_e_ (mg/g)	7.67	2.65	1.03	8.23
	R^2^	0.987	0.53	0.99	0.85
Intraparticle diffusion	K_ipd_ (mg/g min^1/2^)	0.798	0.171	0.03	0.08
	C (mg/g)	0.100	0.091	0.62	7.18
	R^2^	0.840	0.91	0.90	0.74
References	Present study	^29^	^48^	^49^	

### Adsorption isotherm

Data from the investigation were analyzed to identify the most appropriate adsorption isotherm that describes the adsorption process on the chosen bio-adsorbents. The adsorption mechanisms for the Langmuir, Freundlich, and Tempkin isotherms were evaluated using Eqs. 5–7 to fit the data. The optimal isotherm was determined based on the regression coefficient (R) for the linear fit. The results of the isotherm analyses are illustrated in Fig. 12a–c. The Langmuir isotherm (R = 0.999) and pseudo-second-order kinetics (R = 0.987) demonstrated the best alignment with the experimental data. Comparable coefficient values have been reported in other studies^50^. Therefore, we interpreted the adsorption process according to the Langmuir model, indicating that chromium was uniformly adsorbed on the adsorbent’s surface.

As shown in Table 7, which compares the equilibrium parameters of various models and their regression coefficients, this study yields superior values compared to prior research. The comparative study of isotherm parameters for corn cob highlights its strong potential as an effective adsorbent material. In the Langmuir model, corn cob exhibits a maximum adsorption capacity (qm) of 515.5 mg/g, significantly surpassing other materials, along with a high k_1_ value of 242.7 l/mg and an R^2^ value of 0.999, indicating robust adsorption. The Freundlich model further supports these findings with a k_f_ value of 997.7 mg/g and an R^2^ of 0.997, suggesting excellent adsorption characteristics. Additionally, the Temkin model shows favorable parameters, including a bT value of 0.298 j/mol and KT of 0.0007 l/g, with an R^2^ of 0.902. Generally, these results outline the promising potential of corn cob for the removal of contaminants from aqueous solutions.

**Table 7:** Comparative studies of the isotherm parameters derived from corn cob.

Isotherm model	Parameters	Corn cob	Dry bean pod husk	Bentonite	Sewage sludge
Langmuir	K_1_ (l/mg)	242.7	0.032	0.027	0.172
	q_m_(mg/g)	515.5	121.16	0.083	329.8
	R^2^	0.999	0.992	0.5	0.97
Freundlich	k_f_ (mg/g)	997.7	9.88	1.4	96.93
	1/n	0.989	0.51	0.55	0.27
	R^2^	0.997	0.98	0.32	0.87
Temkin	b_T_ (j/mol)	0.298	0.345	6.1	39.64
	K_T_(l/g)	0.0007	0.008	0.24	2.63
	R^2^	0.902	0.75	0.2	0.94
References		Present study	^51^	^52^	^53^

### Regeneration and disposal

Regeneration of CCAC: The CCAC adsorbent demonstrates potential for regeneration using established methods such as acid washing (0.1 M HCl) or thermal treatment (400–600 °C under N_2_), commonly achieving 4–6 adsorption–desorption cycles with < 15% capacity loss for Cr(VI)^24^. Preliminary tests (future work) will quantify cycle stability.

Disposal of Cr-loaded CCAC: Post-use adsorbent poses low leaching risk if stabilized; TCLP tests recommended approving nonhazardous status per EPA. Safe options include secured landfill or co-incineration for Cr recovery.

From an economic perspective, the use of locally sourced agricultural waste as precursor material may reduce raw material costs compared to commercial activated carbon. Although a detailed techno-economic assessment was not conducted in this study, the adsorption capacity achieved under real wastewater conditions is comparable to or higher than several previously reported adsorbents. Future work should include pilot scale validation and cost benefit analysis to assess large scale feasibility.

### Scaling considerations

The lab-scale batch achieved 99.45% Cr(VI) removal from real MAA wastewater (75 min contact time, pH 6.5, 0.175 g/L dosage, 200 rpm. For industrial application, continuous fixed-bed column systems are essential but present distinct challenges compared to batch operation. In fixed-bed carbon columns, one common practical issue is channeling, where water does not flow evenly through the bed but instead forms preferential pathways, reducing effective contact between the adsorbate and the carbon surface. This lowers overall removal efficiency compared to ideal conditions. The problem can be minimized by using graded carbon particle sizes in the range of 0.5–2 mm, which improves packing uniformity and promotes better flow distribution. Another limitation is the reduced contact time in continuous systems: typical empty bed contact times (EBCT) are about 10–30 min, whereas batch systems allow much longer or effectively unlimited contact, often leading to higher equilibrium removal. To address these challenges, future pilot testing is planned at MAA Garment Factory with a treatment capacity of 1–10 m3/day, using multi-stage adsorption columns designed with optimized bed depth and controlled flow rates to enhance performance and maximize adsorption efficiency under real operating conditions.

## Conclusion

In this study, the ultimate and proximate analyses of corn cob were conducted, yielding results such as moisture content (7.95%), volatile matter (75.24%), ash content (3.76%), and fixed carbon (13.05%). The ultimate analysis revealed the elemental composition, including carbon (51.21%), hydrogen (5.49%), nitrogen (0.3%), and oxygen (43%). The activated carbon derived from corn cobs has demonstrated significant effectiveness in extracting chromium from wastewater generated by the MAA Garment Textile Factory. Experimental results conducted at ambient temperature revealed that this corn cob-activated carbon achieved a remarkable chromium removal rate of 99.45% within 75 min, utilizing a pH of 6.5 and a dosage of 0.175 g while stirring at 200 revolutions per minute (rpm). The equilibrium adsorption capacity was recorded at 7.67 mg/g. The chromium adsorption characteristics of this activated carbon were effectively modeled using the Langmuir isotherm, indicating a maximum adsorption capacity of 515.5 mg/g at the same stirring speed. Moreover, both the pseudo-second-order model and the Langmuir model accurately depicted the kinetics and isotherm behaviors observed during the experiments. These findings underscore the potential of corncob-derived activated carbon as a viable and sustainable adsorbent for chromium recovery in industrial wastewater treatment processes. The high removal efficiency not only highlights its effectiveness but also positions it as an economically advantageous alternative to conventional adsorbents, which are often costly and less efficient. In conclusion, this research contributes significantly to the field of environmental management by providing a practical solution to mitigate chromium pollution a pressing concern for industries like textile manufacturing. The successful application of corncob-derived activated carbon not only addresses environmental challenges but also promotes resource recovery, aligning with sustainable industrial practices. Future research should focus on scaling up this technology for broader industrial applications, techno cost analysis and exploring other agricultural by-products for activated carbon production. This approach could further enhance sustainability efforts in wastewater treatment while contributing to circular economy principles.

## Data Availability

The data used and analyzed during this study are available from the corresponding author upon reasonable request.
